# Supplementary material for: Clostridium butyricum Can Promote Bone Development by Regulating Lymphocyte Function in Layer Pullets
Source: Int J Mol Sci. 2023 Jan 11;24(2):1457. doi: 10.3390/ijms24021457 (PMC9867449; doi:10.3390/ijms24021457)
Supplement: Supplementary file 1 [file ijms-24-01457-s001.zip › ijms-2164123-supplementary.pdf]

International Journal of Molecular Sciences

MDPI

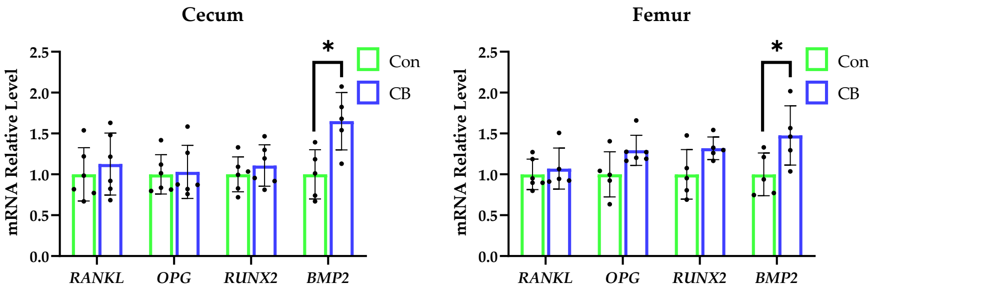

Figure S1. Expression of bone metabolism-related cytokines in cecum and femur. Relative mRNA expression levels of *RANKL*, *OPG*, *RUNX2* and *BMP2* in cecum and femur. Con: normal saline; CB: *Clostridium butyricum*. The data are presented as the mean ±SD. *, $p < 0.05$.

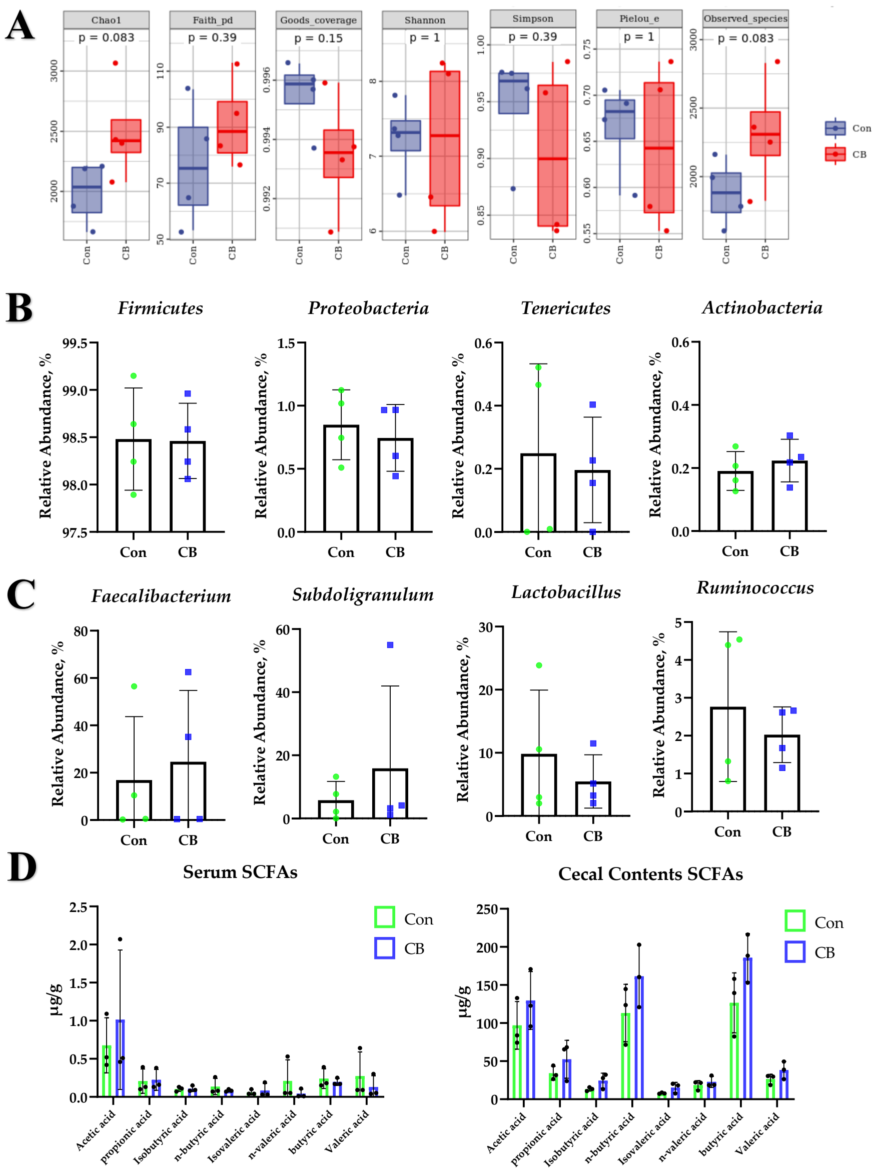

Figure S2. The effect of CB on the gut microbiota. (A) Alpha diversity (Ace, Chao1, Shannon and Simpson). (B) Relative abundance of the top four phylum-level abundances of microbial species in

the cecum. (C) Relative abundance of the top four species-level abundances of microbial species in the cecum. (D) The concentrations of short-chain fatty acids (SCFA) in the serum and cecal contents of the layer pullets. Con: normal saline; CB: *Clostridium butyricum*. The data are presented as the mean ±SD.

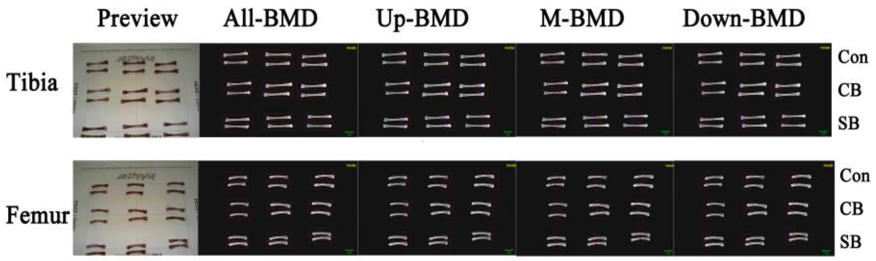

Figure S3. Detection strategy for detecting bone density at different locations. The bone density test defines the upper 1/4 of the bone as the Up-BMD, the lower 1/4 as the Down-BMD, and the middle 1/2 as the M-BMD. Con: normal saline; CB: *Clostridium butyricum*.

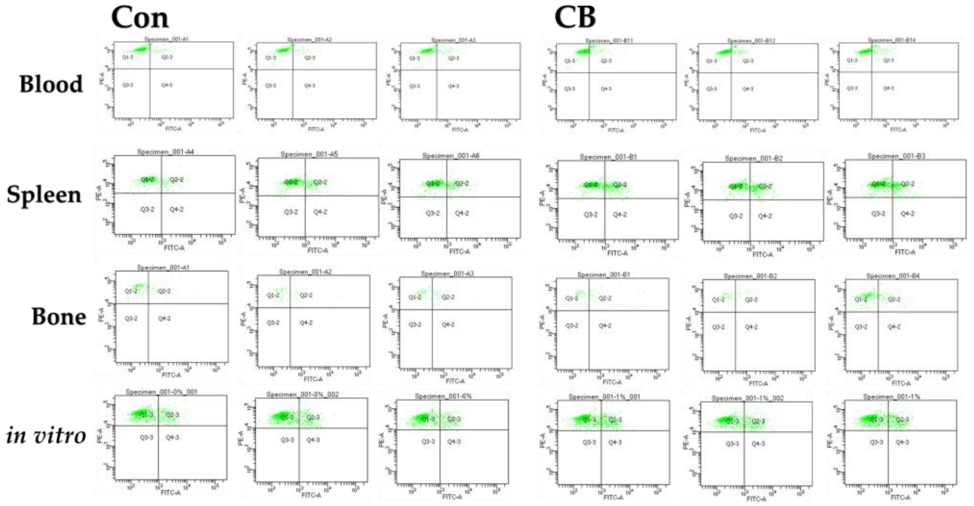

Figure S4. The ratio of $CD3^{+}CD4^{+}CD25^{+}$T cells in peripheral blood, spleen, bone marrow and lymphocytes cultured *in vitro*. Con: normal saline; CB: *Clostridium butyricum*.
